# A Spectrum of Histomorphological and Immunohistochemical Expression Profiles of S-100, CD56 and Calretinin in Benign Peripheral Nerve Sheath Tumours

**DOI:** 10.7759/cureus.40751

**Published:** 2023-06-21

**Authors:** Poornima Jaiswal, Anand CD, Jaison Jacob John

**Affiliations:** 1 Department of Pathology, SRM Medical College Hospital and Research Centre, Chennai, IND

**Keywords:** ihc, immunohistochemistry, calretinin, cd56, s-100, peripheral nerve sheath, neurofibroma, schwannoma

## Abstract

Introduction: Peripheral nerve sheath tumours comprise benign tumours; namely schwannomas and neurofibromas, and only rarely comprise hybrid benign tumours and their malignant counterpart, malignant peripheral nerve sheath tumours (MPNST). There may be diagnostic difficulties in histopathology analysis, especially in core needle biopsies where there is a limited amount of tissue. Immunohistochemistry (IHC) can play a beneficial role, especially in atypical and cellular histological variants and rarely hybrid tumours.

Methods: A total of 45 cases of benign peripheral nerve sheath tumours were included in the study; there were 27 cases of neurofibroma including variants like plexiform and cellular neurofibromas and 18 cases of schwannomas including variants like ancient schwannoma and cellular schwannoma. Immunohistochemical staining (IHC) on these tumour tissues using S-100, CD56 and calretinin was done and scoring was done based on extent and intensity.

Results and discussion: No significant differences were observed between neurofibromas and schwannomas on patient age and anatomical locations of these tumours. IHC results did not show statistically significant patterns of expression of S-100 protein between the schwannoma and neurofibromas groups (p=0.75). CD56 protein was expressed strongly (3+) in 90% of cases of schwannoma and negative in 86% of neurofibromas, the differential expression between the two groups was found to be statistically significant (p <0.0001). Calretinin was positive in 39% of schwannomas including one case of cellular schwannoma and negative in all (100%) cases of neurofibroma while the differential expression of calretinin between schwannoma and neurofibroma groups was found to be statistically significant (p < 0.005).

Conclusion: Our study shows that S-100 does not show differential expression between schwannomas and neurofibromas. CD56 could be a potentially useful IHC marker to aid in the diagnosis of peripheral nerve sheath tumours with significantly higher expression in schwannomas compared to neurofibromas. Calretinin was also found to be preferentially expressed in schwannomas, though the difference is statistically significantly lower compared to CD56. A panel of all these markers could be used for accurate diagnosis.

## Introduction

Peripheral nerve sheath tumours comprise benign tumours, namely schwannomas and neurofibromas and their malignant counterpart, malignant peripheral nerve sheath tumours (MPNST). Schwannomas are composed of immature Schwann cells derived from the peripheral nerve sheath, whereas neurofibromas are composed of Schwann cells and axons, perineurial cells, blood vessels and fibroblasts and mast cells [1-2].

Recent approaches in the diagnosis and management of soft tissue tumours include core needle biopsy analysis for initial diagnosis followed by neoadjuvant chemotherapy in case of malignant lesions (sarcomas) and reducing the size of the tumour, followed by chemotherapy. The type of surgery planned may also depend on the cell of origin and type of tumour, e.g. nerve-sparing surgery, nerve resection and /or nerve repair requirement etc.

An inherent limitation of core needle biopsies is that there will be a limited amount of tissue which may not be representative of the entire lesion which may be heterogenous nature, e.g., alternating hypercellular and hypocellular lesions, varying grades of nuclear atypia in different parts of the tumour and can even affect the grade, especially between intermediate grade and malignant tumours [3].

Some schwannomas with predominantly Antoni B areas, sparse cellularity and/ or large myxomatous areas may resemble a neurofibroma. Some schwannomas may not have palisading nuclei and verocay bodies and may resemble a cellular neurofibroma. Cellular schwannomas may resemble low-grade MPNST and leiomyosarcoma. Plexiform schwannomas are usually devoid of a capsule and composed predominantly of cellular Antoni A areas; when they exhibit increased cellularity and mitotic figures, they may resemble MPNST. Most epithelioid MPNST arise from pre-existing schwannoma [3-5].

The distinction between low-grade MPNST or hypocellular areas of the tumour from atypical neurofibroma and cellular neurofibroma may also be difficult. Neurofibromas exhibiting degenerative atypia and increased cellularity can be mistaken for MPNST [6]. Neurofibromas with ancient change have degenerative atypia but do not show increased cellularity, fascicles and mitotic figures. Neurofibroma can also sometimes mimic dermatofibrosarcoma protruberans (DFSP). Plexiform neurofibromas can also progress to MPNST. MPNST may also have morphological overlap with fibrosarcoma, leiomyosarcoma, dedifferentiated liposarcoma and monophasic synovial sarcoma. Rarely, MPNST can also show glandular differentiation and can mimic a biphasic synovial sarcoma. Hence morphological features alone may not be adequate (especially core needle biopsies) to render a definitive diagnosis in soft tissue tumours and immunohistochemical (IHC) markers (especially a panel of markers) have been found to play a major role to resolve these diagnostic dilemmas [7-12]. The differential expressions of various IHC markers among a spectrum of peripheral nerve sheath tumours have been reported [13-20].

In the present study, we have evaluated the histomorphological features and immunohistochemical expression profiles of S-100, CD 56 and calretinin in peripheral nerve sheath tumours and correlated any difference in morphological features with variations in IHC expression profiles.

## Materials and methods

The study was a retrospective cross-sectional study carried out at the Department of Pathology. Institutional Scientific and Ethical Committee clearance (1998/EIC/2020) was obtained. Cases representing peripheral nerve sheath tumours received in the department during the period from August 2019 to August 2021 were included in the study. A total of 45 cases collected in the above study period were included in the study.

The inclusion criteria were (i) cases representing peripheral nerve sheath tumours received in the histopathology section; (ii) both biopsy and resected specimens of clinically diagnosed cases; and (iii) available basic clinical details including age, gender, and location of tumours.

Exclusion criteria were (i) cases for which overall tissue material is inadequate for immunohistochemical analysis; and (ii) biopsy cases where representative areas were not available.

Clinical data including age, gender, location of the tumour, clinical presentation (whether there was the presence of neurofibromatosis), family history of similar tumours etc., were obtained from the case records.

Hematoxylin and eosin (H & E)-stained microscopic slides of both resection specimens and biopsies were analysed and morphological features were noted and a diagnosis was rendered along with histological variants and special morphological features.

Immunohistochemistry (IHC) involved the peroxidase-antiperoxidase system. Tissue sections (4-5 µ) were made on a rotary microtome (RM2125 RTS, Leica, Wetzlar, Germany). Positively charged slides (PS-011-72, PathnSitu, Secunderabad, India) were used for taking the sections for the IHC procedure. Antigen retrieval on tissue sections was done by microwave method (800W X 7 Minutes, 640 W X 7, 640W X 7 minutes) (MC32K7056CK, Samsung India, Gurugram, India).

Immunohistochemical staining was done on sections using the following primary and secondary antibody detection system:

The primary antibody detection system for IHC from PathnSitu are as follows (a) S-100 beta (Rabbit Monoclonal; Clone EP32; Catalog No PR070-3ml); (b) CD56 (Mouse Monoclonal; Clone 123C3; Catalog No. PM085-3ml); (c) Calretinin (Rabbit Polyclonal, Catalog No. PP134-3ml).

The secondary antibody and detection system from PathnSitu are as follows: poly-excel HRP (horseradish peroxidase) /DAB (3,3’-diaminobenzidine) detection system (PEH002-6ml, Two Step, PathnSitu).

Statistical analysis

Statistical analysis was done using SPSS software v 24.0 (IBM Corp., Armonk, NY). The two-tailed Student t-test method was used to determine the statistical significance between the two groups and the corresponding p-value. A p-value of <0.05 was found to be significant.

## Results

The study included a total of 45 benign peripheral nerve sheath tumours: 18 cases were schwannoma including histological variants, and 27 cases were neurofibroma including histological variants.

The age distribution of the participants and the anatomical location of schwannomas are summarized in Table 1. 

**Table 1:** Age and gender of the participants as well as the anatomical location of schwannomas

S. No	AGE	SEX	SITE	Histopathological Diagnosis
1	13	F	Forearm	Schwannoma
2	19	M	Leg	Schwannoma
3	40	F	Supraclavicular region	Schwannoma
4	65	F	Forearm	Schwannoma
5	57	F	Paravertebral region	Schwannoma
6	40	F	Intramedullary	Schwannoma
7	45	F	Paravertebral region	Schwannoma
8	56	F	Wrist	Schwannoma
9	37	F	Left arm and left knee	Schwannoma
10	46	M	Back	Schwannoma
11	51	M	Neck	Schwannoma
12	23	M	Leg	Schwannoma
13	34	M	Scalp	Schwannoma
14	55	F	Calf	Ancient Schwannoma
15	72	F	Wrist	Ancient Schwannoma
16	51	M	Cervical region	Ancient Schwannoma
17	34	M	Arm	Cellular Schwannoma
18	27	F	Thumb	Cellular Schwannoma

The age of patients ranges from 13-72 years with a median age of 42.5 years. The anatomical locations for schwannomas were as follows: head and neck (n= 5), trunk (n=3), back (n=1), upper limb (n =7), and lower limb (n=2).

**Figure 1:**
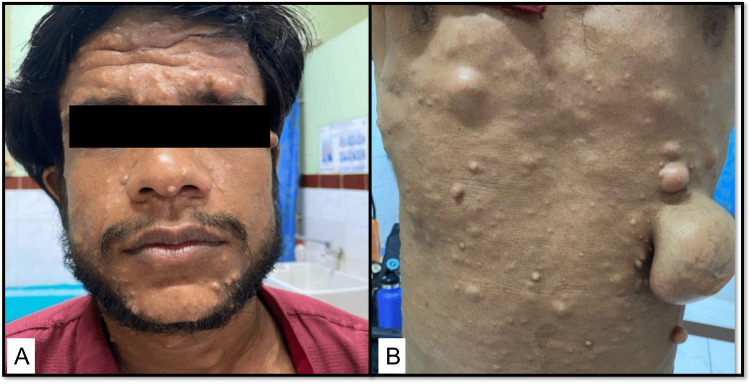
Clinical presentation of a patient with neurofibromatosis 1A: Multiple nodules over the face; 1B: Nodules of varying sizes over the hand

The age distribution and anatomical location of neurofibromas are summarized in Table 2. The age of patients ranged from 14-76 years with a median age of 46 years. The anatomical locations for neurofibromas were as follows: head and neck (n= 9), trunk (n=2), back (n=8), mediastinum (n=1), upper limbs (n=5), and lower limbs (n= 3). One of the patients was a 20-year-old patient with diffuse neurofibromatosis with multiple nodules all over the body (Figure 1).

The age distribution and anatomical location of neurofibromas are summarized in Table 2.

**Table 2:** Age and gender of the participants as well as the anatomical location of neurofibromas

S. No	AGE	SEX	SITE	Histopathological Diagnosis
1	14	F	Scalp	Neurofibroma
2	54	M	Back	Neurofibroma
3	69	M	Back	Neurofibroma
4	70	F	Ear lobe	Neurofibroma
5	63	F	Back	Neurofibroma
6	65	F	Forehead	Neurofibroma
7	21	M	Back	Neurofibroma
8	29	M	Paravertebral region	Neurofibroma
9	33	F	Back	Neurofibroma
10	76	M	Back	Neurofibroma
11	48	F	Hand	Neurofibroma
12	29	M	Chest wall	Neurofibroma
13	37	F	Forehead	Neurofibroma
14	51	F	Index finger	Neurofibroma
15	22	M	Back	Neurofibroma
16	46	M	Forehead	Neurofibroma
17	55	M	Back	Neurofibroma
18	55	F	Mediastinum	Neurofibroma
19	50	M	Knee	Neurofibroma
20	67	M	Cheek	Cellular Neurofibroma
21	20	F	Neck	Cellular Neurofibroma
22	25	M	Foot	Neurofibroma
23	49	F	Scalp	Neurofibroma
24	27	F	Leg	Neurofibroma
25	25	F	Forearm	Neurofibroma
26	40	F	Neck	Plexiform Neurofibroma
27	44	F	Arm	Plexiform Neurofibroma

The histomorphological features of cases diagnosed with schwannoma and neurofibroma with histological variants and distributions of schwannoma cases with the proportion of Antonia A and Antoni B areas are summarized in Table 3.

**Table 3:** Unique histological features and variants in benign peripheral nerve sheath tumours

SCHWANNOMA (n= 18)		NEUROFIBROMA (n=27)	
Both Antoni A and Antoni B areas	11	Diffuse neurofibroma	22
Only Antoni A areas	2	Myxoid change	1
Only Antoni B	5		
Histological variants		Histological variants	
Cellular schwannoma	2	Plexiform neurofibroma	2
Ancient schwannoma	3	Cellular neurofibroma	2

**Figure 2:**
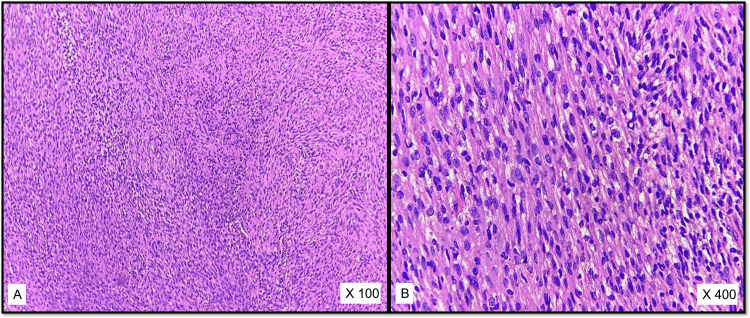
A, B: Cellular Schwannoma (H & E)

Out of 18 cases diagnosed as schwannoma by histopathology, 11 cases had both Antoni A and B areas whereas six cases had only Antoni B areas with hypocellular, reticular, microcystic areas and lacking palisading nuclei. Two cases of cellular schwannomas (Figures 2A-2B) and three cases of ancient schwannoma were reported.

**Figure 3:**
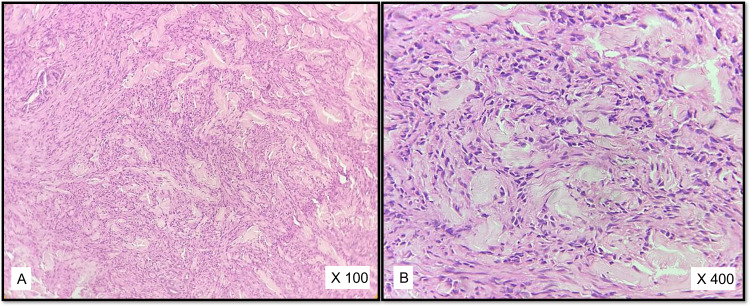
A, B: Cellular neurofibroma (H& E)

Out of 27 cases diagnosed as neurofibroma by histopathology, 22 cases were of the diffuse type, two of plexiform neurofibroma, one showing extensive myxoid change and two of cellular neurofibroma (Figures 3A-3B).

Immunohistochemistry (IHC) results

The IHC results (S-100, CD 56 and calretinin) were recorded as per the scoring criteria in Table 4. 

**Table 4:** Immunohistochemistry (IHC): scoring system for S100, CD56 and calretinin An objective scoring system measuring the extent of staining within the tumour compartment as well as the intensity of staining was used.

Extent of the staining within tumour cells	
<25%	1+
25% to 75%	2+
>75%	3+
Intensity of staining	
Weak	1+
Moderate	2+
Strong	3+

IHC expression profiles of all three markers for neurofibromas and schwannomas are summarized in Tables 5-6 respectively.

**Table 5:** IHC results (S-100, CD56 and calretinin) in neurofibromas

Sl No	HISTOPATHOLOGY DIAGNOSIS	S100 (nuclear & cytoplasmic)		CD56 (cytoplasmic)		CALRETININ (cytoplasmic)	
		Extent	Intensity	Extent	Intensity	Extent	Intensity
2	Neurofibroma	+	3+	+	2+	0	-
3	Neurofibroma	+	3+	0	-	0	-
4	Neurofibroma	+	3+	0	-	0	-
5	Neurofibroma	+	2+	1+	1+	0	-
6	Neurofibroma	+	3+	0	-	0	-
7	Neurofibroma	+	3+	0	-	0	-
8	neurofibroma	+	3+	0	-	0	-
9	Neurofibroma	+	3+	0	-	0	-
10	Neurofibroma	+	3+	0	-	0	-
11	Neurofibroma	+	3+	0	-	0	-
12	Neurofibroma	+	3+	0	-	0	-
13	Neurofibroma	+	3+	0	-	0	-
14	Neurofibroma	+	3+	0	-	0	-
15	Neurofibroma	+	3+	0	-	0	-
16	Neurofibroma	+	3+	+	3+	0	-
17	Neurofibroma	+	2+	0	-	0	-
18	Neurofibroma	+	3+	0	-	0	-
19	Neurofibroma	+	2+	0	-	0	-
20	Neurofibroma	+	3+	0	-	0	-
21	Neurofibroma	+	3+	+	2+	0	-
22	Neurofibroma	+	2+	0	-	0	-
23	Plexiform neurofibroma	+	2+	0	-	0	-
24	Neurofibroma with myxoid change	+	3+	0	-	0	-
25	Plexiform neurofibroma	+	3+	0	-	0	-
26	Cellular neurofibroma	+	3+	0	-	0	-
27	Cellular neurofibroma	+	3+	0	-	0	-

**Table 6:** IHC results (S-100, CD56 and calretinin) in schwannomas

Sl No	HISTOPATHOLOGY DIAGNOSIS	S-100 (nuclear & cytoplasmic)		CD56 (cytoplasmic)		CALRETININ (cytoplasmic)	
		Extent	Intensity	Extent	Intensity	Extent	Intensity
1	Ancient schwannoma	+	3+	+	3+	1+	1+
2	Ancient schwannoma	+	3+	+	3+	1+	2+
3	Cellular schwannoma	+	3+	+	3+	2+	2+
4	Cellular schwannoma	+	3+	+	3+	0	-
5	Schwannoma	+	2+	+	1+	0	-
6	Schwannoma	+	3+	+	2+	0	-
7	Schwannoma	+	3+	+	2+	0	-
8	Schwannoma	+	3+	+	3+	0	-
9	Schwannoma	+	3+	+	2+	0	-
10	Schwannoma	+	3+	+	3+	0	-
11	Schwannoma	+	3+	+	3+	2+	2+
12	Schwannoma	+	3+	+	3+	0	-
13	Schwannoma	+	3+	+	3+	0	-
14	Schwannoma	+	3+	+	3+	1+	2+
15	Schwannoma	+	3+	+	3+	1+	3+
16	Schwannoma	+	3+	+	3+	1+	2+
17	Schwannoma	+	3+	+	3+	0	-
18	Schwannoma	+	3+	+	3+	0	-

**Figure 4:**
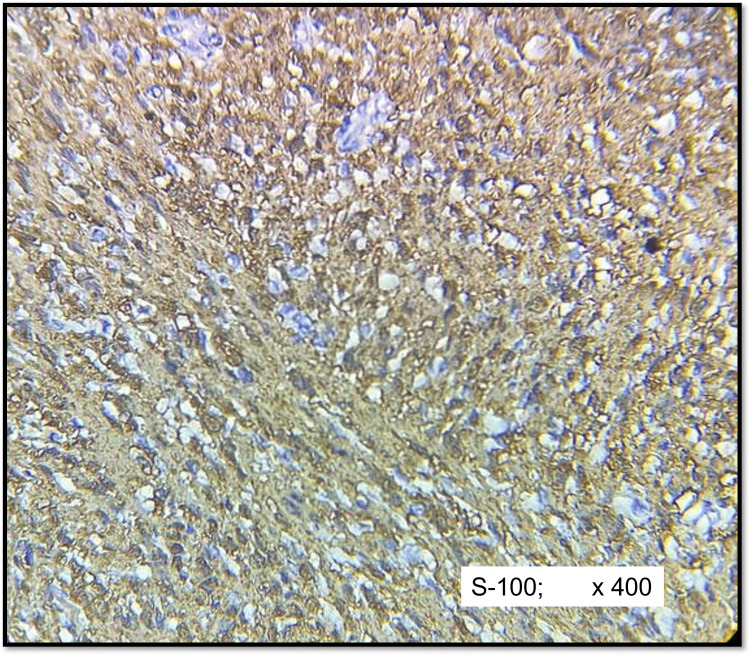
IHC of cellular schwannoma (S-100) Diffuse strong positivity in tumour cells

**Figure 5:**
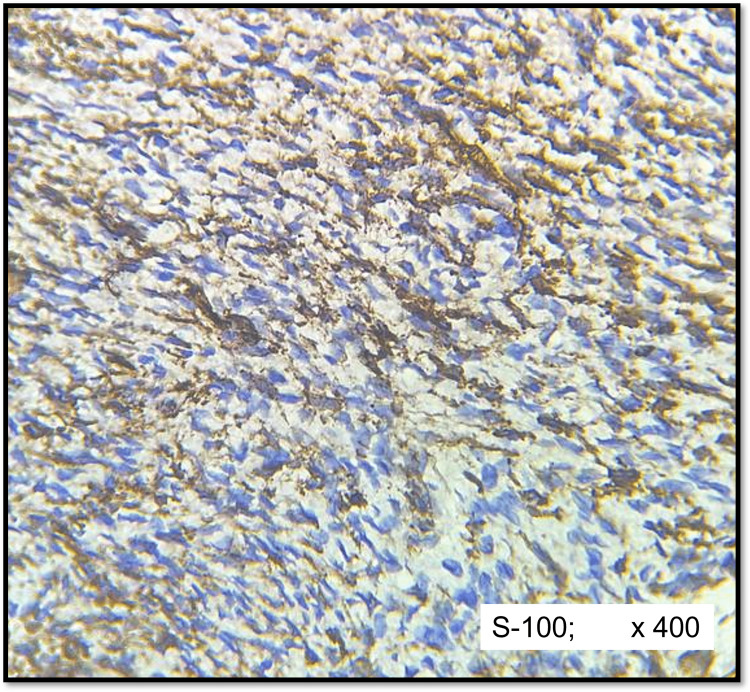
IHC of cellular neurofibroma (S-100) Moderate to strong positivity in tumour cells

IHC results showed diffuse strong positivity (3+) of S-100 in both schwannomas (Figure 4) and neurofibromas (Figure 5) except for four cases (14.2%) of neurofibroma that showed moderate staining intensity (2+). There was no statistically significant pattern of expression of S-100 protein observed between the schwannoma and neurofibromas groups (p =0.75). 

**Figure 6:**
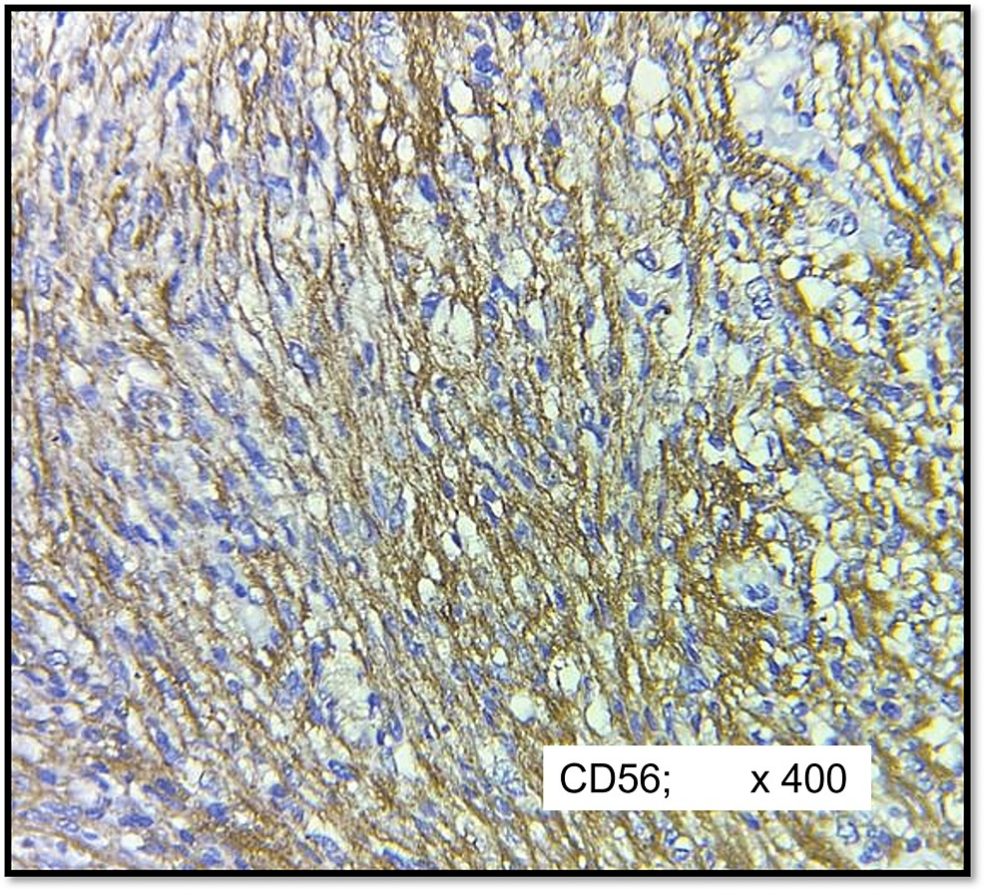
IHC of cellular schwannoma (CD56) Diffuse strong positivity in tumour cells

**Figure 7:**
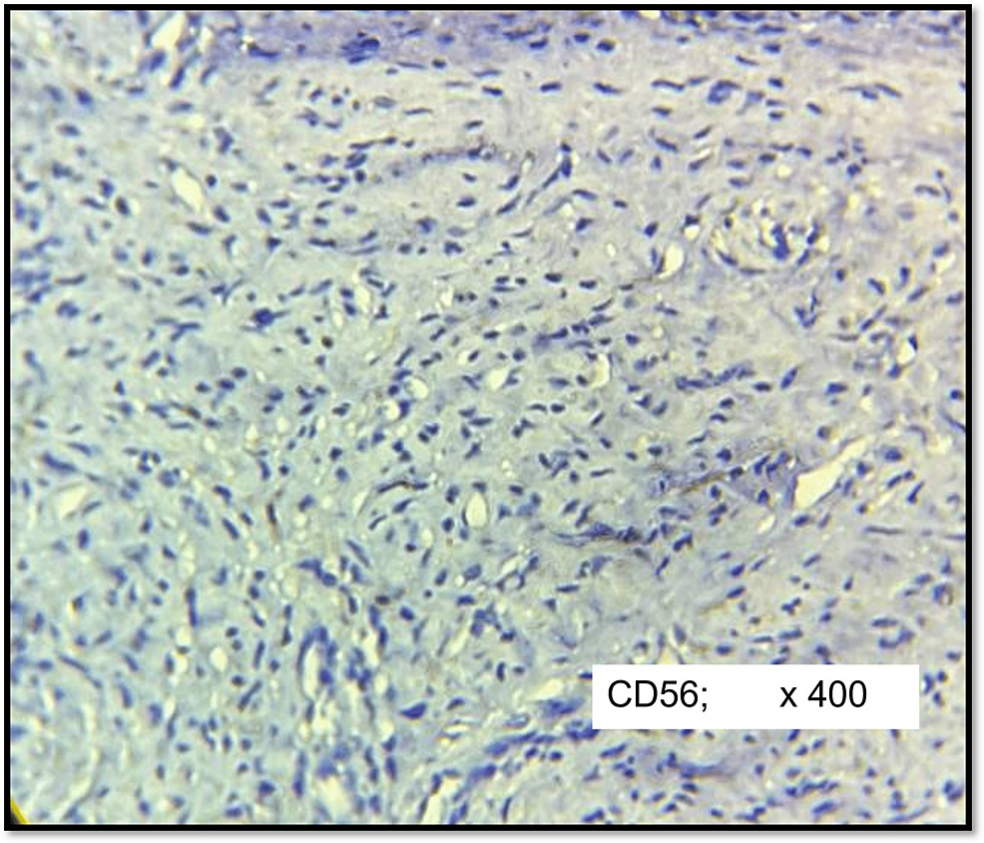
IHC of cellular neurofibroma (CD56) Negative in tumour cells

CD56 was expressed strongly (3+) in 90% (n=16) of cases of schwannoma (Figure 6) except in one case (5%) with moderate positivity (2+) and another case (5%) showing mild positivity (1+). CD 56 was negative in 86% (n=23) of neurofibromas (Figure 7) and two cases (7%) showed mild positivity (1+), one case (3%) with moderate positivity (2+) and one case (3%) with strong positivity (3+). The differential expression of CD56 between schwannoma and neurofibroma groups was found to be statistically significant (p <0.0001).

**Figure 8:**
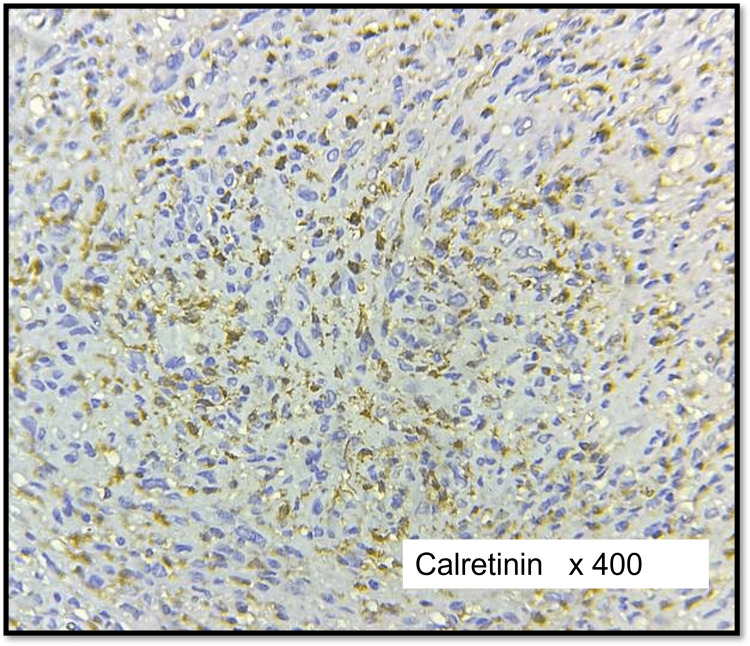
IHC of cellular schwannoma (calretinin) Moderate to strong positivity in tumour cells

**Figure 9:**
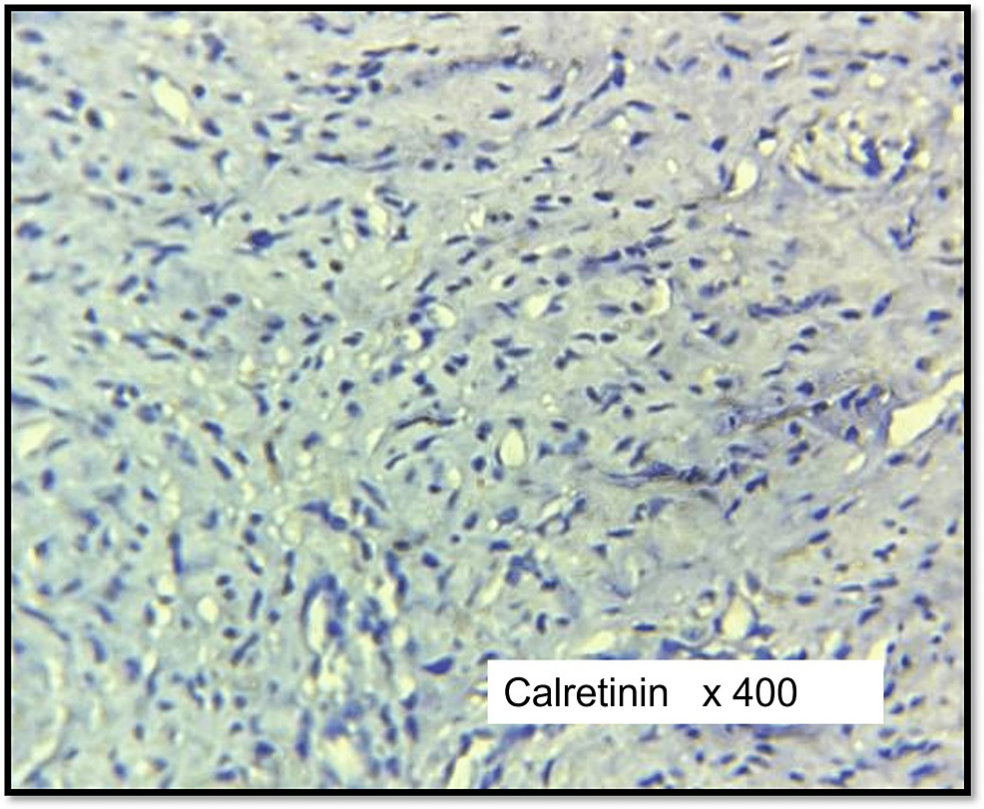
IHC of cellular neurofibroma (calretinin) Negative in tumour cells

Calretinin was positive in seven cases (39%) of schwannomas (Figure 8) including one case of cellular schwannoma and found negative in all cases of neurofibroma (Figure 9). The differential expression of calretinin between schwannoma and neurofibroma groups was found to be statistically significant (p < 0.005).

## Discussion

Clinical parameters

No significant differences were observed between schwannomas on patient age (median age group being 42.5 and 46 yrs) and anatomical distribution (Table 1) that conform to previous literature [2], including Rodriguez et al [3]. The male-to-female ratio was comparatively higher in schwannomas (1.7:1) compared to neurofibroma (0.8: 1) which was consistent with findings in previously published literature.

S-100 

S-100 is a protein seen in neural or neural crest-derived tissues and is also expressed in chondrocytes, melanocytes and Langerhans cells. It is a common marker for neurogenic tumours. S-100 has been reported to be positive in both Schwannomas and neurofibromas, many previous studies including Park et al. [4], Awf Sh et al. [10], Guo et al. [11] reported that they are expressed more diffusely and strongly in schwannomas compared to neurofibromas. S-100 has a patchy, scattered positivity in MPNSTs which differentiates it from other sarcomas.

The present study, however, did not show any statistically significant difference (p=0.75) in staining (extent and intensity) between schwannomas and neurofibromas. Diffuse strong positivity (3+) of S100 in both schwannomas and neurofibromas was observed except for four cases (14.2%) of neurofibromas that showed moderate staining intensity (2+). On S100 staining, more than 75% of cells were positive in cases of cellular neurofibroma, plexiform neurofibroma and diffuse neurofibroma. With the above observations, it is found that S100 expression is maximum in all cases of schwannoma attributing to its origin from Schwann cells while the expression is decreased in neurofibroma as it is composed of a mixed population of cells such as Schwann cells, perineurial-like cells and endoneurial fibroblasts. Staining of tumour cells for S100 was in concordance with the study done by Fine et al [5].

CD56 (Neural Cell Adhesion Molecule)

Park et al. [4], and Guedes-Corrêa et al. [9] have shown that CD56 could be used in combination with S-100 and calretinin in the diagnosis of peripheral nerve sheath tumours and Guo et al. [11] have reported that CD56 shows preferential expression in schwannomas compared to neurofibromas and a slightly lower specificity compared to calretinin.

In the present study, there is a consistency with previous studies on preferential expression in schwannomas but there is a statistically significant difference (p <0.0001) with 100 % negative expression in neurofibromas.

Calretinin

Calretinin is a calcium-binding protein expressed in certain neurons in the central nervous system and peripheral nerves and also in mesothelial cells as reported by Dabbs [12]. Fine et al. [5] have reported the potential use of calretinin preferentially expressed in schwannomas. Park et al. [4] and Guo et al. [11] have found it to be very specific for schwannomas, with nearly a 100% of the cases studied being positive in schwannomas and negative in neurofibromas.

In the present study, calretinin was positive in only seven cases (39%) of schwannomas including one case of cellular schwannoma and negative in all cases (100%) of neurofibromas. The differential expression of calretinin between schwannoma and neurofibroma groups was found to be statistically significant (p < 0.005).

In the present study, CD56 was found to be more significantly and selectively expressed in Schwannomas compared to Calretinin.

Using IHC Results for Resolving Differential Diagnoses on Histopathology

Most of the cases were distinguished based on the histological pattern and cellular components but three cases out of 45 benign peripheral nerve sheath tumours were difficult to differentiate on H&E stained sections due to a lack of nuclear palisading and the presence of only hypercellular areas. Hence, differential diagnoses of cellular neurofibroma and cellular schwannoma were thought of. For these three cases, differentiation was done based on the IHC marker CD56 and calretinin. One out of three cases was positive for CD56 and calretinin and hence, was reported as cellular schwannoma and two of three cases were non-reactive for both CD56 and calretinin. Hence was reported as cellular neurofibroma. All these three cases were positive for S100 diffusely with strong intensity (3+). 

The study has a few limitations and can be followed up with an increased representation of histological variants with morphological overlap and also expand this IHC panel to include newer neural markers including SOX-10 [13,14]. Follow-up of patients with clinical data especially recurrence and malignant transformation could be useful to predict the aggressiveness of the tumour.

## Conclusions

Accurate diagnosis of peripheral nerve sheath tumours is important, especially on core-needle biopsies with limited tissue which may not be completely representative of the tumour. Diagnosis of histological variants including atypical/ cellular neurofibroma, cellular schwannoma, schwannomas lacking palisading nuclei, more hypocellular areas from low-grade malignant peripheral nerve sheath tumour (MPNST), the possibility of hybrid tumours with both components could be challenging on histopathology alone. In such settings, immunohistochemistry has been shown to play a role in arriving at the right diagnosis which may be important to plan the management including the type of surgery (including nerve-sparing techniques) after the histopathology report on the core needle biopsy. Many of the schwannomas may be treated without resection of the nerve while neurofibromas may require partial resection and nerve repair.

Our study shows that S-100 does not show differential expression between schwannomas and neurofibromas. However, CD56 has been found to be a more sensitive marker aid in the diagnosis of peripheral nerve sheath tumours with statistically significantly higher expression in schwannomas compared to neurofibromas. Calretinin was also found to be statistically significantly highly expressed in schwannomas compared to neurofibromas, though the difference is statistically significantly lower compared to CD56. Thus we conclude from our findings that immunohistochemical analysis of CD56 and Calretinin will be a valuable ancillary aid to the histopathologist in rendering the right diagnosis of peripheral nerve sheath tumours, especially in the era of core needle/ small biopsies for soft tissue neoplasms and also when there are histological overlapping features including variants.
